# Editorial for the Special Issue on Industrial Machine Learning Applications

**DOI:** 10.3390/jimaging9120278

**Published:** 2023-12-14

**Authors:** Paolo Rota, Miguel Angel Guevara Lopez, Francesco Setti

**Affiliations:** 1 Center for Mind and Brain (CIMeC), Department of Engineering and Computer Science, University of Trento, 38122 Trento, Italy; 2 Department of Systems and Computing, Setubal School of Technology, Polytechnic Institute of Setubal, Campus do IPS, Estefanilha, 2910-761 Setubal, Portugal; miguel.lopez@estsetubal.ips.pt; 3 Department of Computer Science, University of Verona, 37134 Verona, Italy; francesco.setti@univr.it

In the rapidly evolving field of industrial machine learning, this Special Issue on Industrial Machine Learning Applications aims to shed light on the innovative strides made toward more intelligent, more efficient, and adaptive industrial processes. The contributions within this issue span diverse applications, each showcasing the transformative potential of machine learning when applied to the unique challenges of industrial settings.

As presented in one of the contributions [1], integrating machine learning into industrial robotics exemplifies the synergy between advanced algorithms and physical systems. Developing a visual object detection and localization workflow tailored for the precision required in car door assembly demonstrates the practical benefits of deploying learning-based methods on mobile robotic platforms. This approach not only enhances the capabilities of robots in terms of grasping but also underscores the adaptability of machine learning to the intricacies of manufacturing environments.

Another study delves into the realm of infrastructure maintenance [2], where the optimization of convolutional neural networks plays a pivotal role in detecting pipe bursts within water distribution networks. The meticulous tuning of network parameters to suit the specificities of real-world data highlights the importance of model adaptability. This research contributes to preventing water loss and illustrates the broader applicability of machine learning in resource management and conservation.

The issue also explores the critical aspect of predictive maintenance through a transfer learning-based approach for estimating machinery's Remaining Useful Life (RUL) [3]. By leveraging limited datasets and low sampling rates, the proposed method showcases the power of abstracting features and combining convolutional layers with Long Short-Term Memory networks. This approach not only sets a new benchmark in the field but also emphasizes the value of transfer learning in extending the lifespan of industrial components.

In the domain of materials science, the challenge of training deep convolutional neural networks with limited labeled data is addressed through the generation of synthetic X-ray computed tomography data [4]. The innovative use of data augmentations and a unique network architecture underscores synthetic data's potential to overcome manual labeling constraints. This research achieves impressive segmentation results and sheds light on the broader implications for material characterization and quality control.

Lastly, the enhancement of inspection robots for electrical substations through an improved neural network architecture demonstrates the critical role of machine learning in ensuring the safety and reliability of power infrastructure [5]. The modifications made to the network, aimed at meeting the stringent demands of real-time and accurate inspection, exemplify the continuous pursuit of performance optimization in machine learning applications.

Collectively, the contributions to this Special Issue illustrate the transformative impact of machine learning across various industrial sectors. From robotics and infrastructure to predictive maintenance and materials science, the research presented here provides a glimpse into a future where machine learning is not just a tool but a fundamental component of industrial innovation. As we continue to push the boundaries of what is possible, the Journal of Imaging remains committed to highlighting the cutting-edge work that will shape the industries of tomorrow.
